# Supplementary material for: Are carbon emissions trading and green financial instruments synergistic? -Comprehensive quantitative research based on content analysis
Source: PLoS One. 2024 Mar 7;19(3):e0298601. doi: 10.1371/journal.pone.0298601 (PMC10919601; doi:10.1371/journal.pone.0298601)
Supplement: S1 Data — (ZIP) [file pone.0298601.s001.zip › Data/地级市Z_F工作报告.zip!/╨┬╜«/┐╦└¡┬Ω╥└╩╨/└┤╫╘╣1⁄2╓┌║┼í╛┬φ┐╦╩2╛▌═°í┐_2017.docx]

各位代表：

现在，我代表市人民政府向大会作政府工作报告，请予审议，并请市政协委员和列席人员提出意见。

过去五年的工作回顾

2012年以来的五年，是我市深化改革力度最大、城市转型推进最快、经济社会发展最好、生态环境改善最大、群众得到实惠最多的时期。五年来，在自治区党委和市委的正确领导下，面对经济和社会两个“三期叠加”的复杂形势，市人民政府团结和带领全市人民，坚定打造世界石油城战略目标不动摇，凝心聚力，抢抓机遇，聚焦城市转型和可持续发展，突出创新引领，各项事业取得了积极成效，开创了克拉玛依改革发展稳定的崭新局面。

——五年来，特别是近两年来，面对新常态下经济下行压力加大、国际油价断崖式下跌的严峻挑战，我们出台一系列政策措施，稳增长、促发展，地区生产总值实现年均增长3.3%， 2016年为648.9亿元，保持了平稳发展。扣除中央石油石化企业贡献份额，地方生产总值由104.7亿元增加到176.6亿元（2011年至2016年，下同），年均增速8.7%。第三产业占GDP比重由16.2%提高到28.2%，产业结构进一步优化，发展质量进一步提升。

——公共财政收入从51亿元增加到79.1亿元，年均增速9.2%，为改革发展稳定和改善民生提供了坚实保障。民生支出累计达333.9亿元，占公共财政支出的77.1%；城镇居民人均可支配收入和农村居民人均纯收入年均增长12.6%和9.1%，城镇登记失业率始终保持在1%以内，市民的幸福感进一步增强。

——辖区总人口由43.6万人增加到49.3万人，增长13.1%；引进各类高层次人才494人，其中硕士460人、博士18人、其他高端领军人才16人，城市的吸引力进一步提升。

——人均公园绿地面积由9平方米，增加到11.6平方米，全市空气质量优良率保持在90%以上，人均住房面积由27.7平方米增加到40.4平方米，城市环境更加优美宜居。

——社会大局保持和谐稳定，社会稳定和长治久安的基础更加牢固，连续五年入选“中国最安全城市”排行榜，成为全疆唯一入选的城市。

——市本级行政许可事项减少460余项，行政许可承诺审批时限压缩15%，政府简政放权、放管结合、优化服务持续深入推进，治理体系和治理能力现代化水平进一步提升。

（一）重要领域改革全面深化，经济和社会发展活力显著增强。

五年来，我们坚持问题导向，破解制约经济社会发展的体制机制问题，取得了显著成效。紧紧围绕放管服改革，持续推进行政管理体制改革和政府职能转变，进一步理顺了部门职责关系，行政审批事项大幅压缩，行政效能进一步提升。紧紧围绕发展推进经济领域改革，央企属地注册与合资合作改革取得重大突破，与社会资本合资合作逐步推开。地方企业持续减负，智力资源流通进一步畅通，经济发展活力不断增强。紧紧围绕群众期盼推进社会领域改革，公立医院改革加快推进，现代学校制度和管理育人模式基本建立，各族群众的获得感进一步增强。

（二）产业结构不断优化升级，经济发展质量和效益显著提高。

以六大基地为核心的石油石化主业平稳发展，产业链进一步延伸。五年来，累计生产原油5736.1万吨，加工原油6905.5万吨，生产天然气156.5亿方，生产乙烯627.4万吨。黑油山、富城能源等企业参与油气开发规模不断扩大。克石化公司实现属地法人注册，对地方经济发展贡献持续增强。成立了新疆石油工程技术服务企业联合会，设立了新疆石油工程技术服务企业资质认定管理中心，技术服务实力进一步增强。宝鸡钢管、江苏金石等一批装备制造项目建成投产。中国石油大学（北京）克拉玛依校区成为新疆首所教育部直属高等院校，建成了国内最大的石油石化全产业链实训基地，服务“一带一路”建设和国家能源战略的能力进一步提升。

以三大新兴产业为先导的第三产业较快发展，经济发展活力进一步增强。五年新增各类金融机构66家，初步形成了银行、保险、证券、期货、担保、股权投资等业态齐全的金融服务体系。中亚商品交易中心成为国内首家成品油在线销售平台。资本市场建设取得重大突破，贝肯能源在A股市场成功上市，19家企业在新三板挂牌。建成了全国首个“三网融合”城市，在全疆首家开通4G网络服务。云计算产业园被列为国家重大工程，吸引了60余家企业（项目）落户，园区数据中心规模居全国前列。旅游业形成石油工业、地质地貌和城市休闲等特色业态，成为首批国家级旅游业改革创新先行区。

在以供给侧结构性改革推进传统产业优化升级和新兴产业创新发展的同时，以“大众创业、万众创新”为新引擎的新业态也得到较快发展，商贸物流、现代服务、健康养老、文化体育等产业呈多元发展态势，民营经济、个体经济得到长足发展，市场活力进一步增强。突出成果转化，依托驻市央企勘探开发、炼油化工、实验检测等科技资源，引入大连化物所，强化地方研究机构作用，为科技成果转化和创新发展提供了有力支撑。

（三）对外交流合作日益扩大，城市吸引力和影响力显著增强。

积极把克拉玛依的发展融入国家“一带一路”倡议，加快构建对外开放的新格局，先后与亚欧、北美等国家的15个城市建立了友好联系，与俄罗斯、哈萨克斯坦、巴基斯坦、蒙古的5个城市缔结为友好城市，加入世界能源城市伙伴组织（WECP），具备了良好的国际交流合作基础，国际化、现代化水平显著提升。积极打造开放合作平台，策划举办了丝绸之路经济带新疆·克拉玛依论坛、丝绸之路科学节、中国（克拉玛依）国际石油天然气及石化技术装备展览会、信息化创新克拉玛依国际学术论坛等国际性展会活动，务实推进与国内及丝绸之路沿线各国互联互通、经贸合作、人文交流，城市影响力不断扩大。区域交通枢纽初步形成，克拉玛依—塔城、克拉玛依—阿勒泰高速公路竣工通车，克拉玛依—乌鲁木齐城际列车开通运行，新增往返北京、上海、郑州、武汉、成都等多条航线，公路、铁路、航空三位一体的立体交通格局逐步建立。区域中心城市建设成效显现，北疆西部区域融合发展格局初步形成，与兵团第七师共建的“兵地油融合发展试验区”成为自治区区域经济发展的样板地区。

（四）城市建设力度持续加大，现代化和高品质水平显著提高。

城市总体规划修编全面完成，中心城建成区面积扩大了一倍。五年累计投入249.4亿元，高质量、高水平完成了会展中心、文体中心、工程教育基地、厚博学院（一期）、残疾人综合服务中心等380余项工程建设，城市功能日趋完善，城市面貌日新月异，城市品质大幅提升。生态文明建设扎实推进，实施了“大绿化”工程和“蓝天工程”，完成了金龙湖应急水源、古海湿地公园（一期）等生态工程建设，中心城区已基本形成一条河、一片湿地、四片森林、六个湖泊的生态环境体系，五年累计新增绿地7.4万亩，基本形成“把城市建在森林中”的生态格局。建立了城市管理“大联动”体制机制与精细化管理标准考核体系，城市精细化管理水平显著提升。将诚信体系建设与城市管理相结合，建立了覆盖全社会的守信激励和失信惩戒机制，我市被确定为自治区信用体系建设综合试验区。文明城市创建取得突破，荣获并蝉联“全国文明城市”称号。

（五）各项社会事业蓬勃发展，市民生活质量和水平显著提高。

持续推进“十大民生工程”建设，民生福祉得到持续改善，一批事关群众切身利益的问题得到有效解决。住房条件明显改善，五年累计开工建设各类保障性住房3.1万套，人均住房面积增长45.8%。千方百计促进就业，出台“双创”等积极的就业创业政策，高校毕业生年均就业率85%以上，经济发展与扩大就业联动机制的促进作用明显增强。社会保障体系不断完善，连续5年提高基本养老金、城乡居民最低生活保障标准，3次增调最低工资标准，在全疆率先实现城乡居民基本养老和医疗保险一体化，居家养老服务模式成为全国样板。教育事业取得长足发展，与国内外5所中学、大学合作办学，丰富了教育教学资源，提升了办学特色。连续5年普通高考综合录取率保持在95%以上，荣获基础教育国家级教学成果特等奖，在全疆率先基本实现教育现代化。医疗卫生事业快速发展，实施公立医院改革，取消药品加成，药品价格平均降幅13%，与国内外32家知名医院建立合作关系，让市民享受优质医疗资源。成功创建国家公共文化服务体系示范区，文化馆、科技馆相继建成投用，成功举办自治区第十三届全运会、新疆国际民族舞蹈节、国际马拉松赛等一批大型文体赛事活动，群众多层次、多元化的文体需求得到满足。

（六）社会大局保持和谐稳定，实现长治久安的基础更加牢固。

紧紧围绕社会稳定和长治久安的总目标，有效促进了各族群众共居共学共乐，进一步夯实了民族团结的情感基础。在全国首推个人诉讼保险制度，鼓励引导群众通过法律渠道表达利益诉求，社会治理能力进一步提升，公众安全感进一步增强。

各位代表，回顾五年的奋斗历程，我们得到许多有益启示，也为今后发展积累了宝贵经验：一是必须坚持在市委的坚强领导下，牢固树立政治意识、大局意识、核心意识、看齐意识，不打折扣地把中央、自治区和市委的各项决策部署落实到位。二是必须围绕社会稳定和长治久安的总目标，正确把握稳定和发展这两个关键点，始终坚持稳定压倒一切，以稳定促发展，以发展保稳定。三是必须增强战略定力，坚定打造世界石油城的战略目标不动摇，一张蓝图绘到底，一以贯之抓好落实，持续推进城市科学转型。四是必须强化创新意识、开放意识、融合共享意识，面向世界学习、面向未来发展，勇于开拓创新，敢想、敢干、敢为人先，开创具有克拉玛依特色的转型发展之路。五是必须坚持以人民为本、民生优先，让全市人民共享改革发展成果，使各族群众有更多的获得感，凝民心、聚民力、集民智，努力建设团结和谐、繁荣富裕、文明进步、安居乐业的克拉玛依。六是必须传承和弘扬克拉玛依精神，坚持全市一盘棋、一家人、一条心、一起干的优良传统，同举克拉玛依旗、共创克拉玛依业，地企同心合力、各方团结一致，共同把我们生活的家园建设得更加美好。

各位代表，五年来的发展成就来之不易、令人鼓舞。这些成绩的取得，是自治区党委、自治区人民政府关心支持和市委正确领导的结果，是全市人民开拓进取、真抓实干的结果，是市人大及其常委会有效监督、市政协积极参政议政的结果，是驻市中央企业与地方发挥体制优势，步调一致谋划发展、团结一心推动发展的结果，是所有在克拉玛依投资置业、务工经商人士，以及关心支持克拉玛依发展建设的驻市各单位、兵团团场、社会团体、友好人士、离退休老同志共同努力的结果，是驻市人民解放军、武警官兵，全市政法干警、广大民兵无私奉献的结果。在此，我代表市人民政府表示最诚挚的感谢和最崇高的敬意！

总结过去，我们也清醒地看到，在前进的道路上，我市正面临着发展与转型的双重任务、促进经济增长与维护社会稳定的双重压力，经济社会发展仍存在不少困难和问题，主要表现在：一是部分发展指标没有实现预期目标，产业结构单一的局面仍未得到根本改变，传统产业优化升级和新兴产业创新发展步伐还不够快，产业多元化发展仍需加倍努力。二是地方企业创新发展和走出去发展能力不强。三是城市管理、社会治理水平与群众日益增长的需求还有差距，改善民生的任务艰巨复杂。四是政府有些部门办事效率不高，部分公务人员还存在作风不实的问题。对此，我们一定要以高度负责的精神，采取积极有效措施，切实加以解决。

今后五年的工作部署

今后五年，是我们围绕社会稳定和长治久安总目标，加速推进城市科学转型的关键时期。市第十一次党代会提出了增强战略定力，推进科学转型，为实现克拉玛依社会稳定和长治久安而努力奋斗的目标，对进一步推进“632”发展战略作出了全面部署。

按照市委的总体部署和“十三五”规划，今后五年政府工作的指导思想是：高举中国特色社会主义伟大旗帜，以邓小平理论、“三个代表”重要思想、科学发展观为指导，深入贯彻落实习近平总书记系列重要讲话精神、特别是在第二次中央新疆工作座谈会上的重要讲话和视察新疆时的重要讲话精神，紧紧围绕社会稳定和长治久安这个总目标，以“五位一体”总体布局、“四个全面”战略布局和“五大发展理念”为引领，认真贯彻落实自治区第九次党代会和市第十一次党代会精神，深入实施“632”发展战略，为打造世界石油城，实现克拉玛依社会稳定和长治久安而努力奋斗。

今后五年的主要奋斗目标是：

——经济持续稳定发展。到2021年，实现地区生产总值1200亿元，年均增长7%，扣除中央石油石化企业贡献份额，地方生产总值年均增长13%；全社会固定资产投资累计实现2000亿元；一般公共预算收入实现105亿元，年均增长7%；第三产业增加值年均增长12%，占GDP的比重超过35%；非油经济实现增加值350亿元，占GDP的比重超过30%；全社会研究与试验发展经费支出与生产总值的比例超过3.7%；实现进出口贸易额5亿美元，年均增长25%以上。

——城市品质显著提升。创建中国人居环境范例奖。辖区总人口60万人左右，中心城区人口超过45万人；全市绿地面积达到12万亩，人均公园绿地面积达到13.8平方米，环境空气质量优良率91%以上，人均体育场地面积6.5平方米，居全国前列；全面建成智慧城市，居民家庭宽带接入能力达到500兆/秒，市民在教育、医疗、交通等领域享受更加优质高效的服务；蝉联“全国文明城市”称号。

——社会事业全面进步。城镇居民人均可支配收入达到5万元，农牧民人均纯收入达3.3万元；办成极具特色和有吸引力的一流教育，适龄青年高等教育入学率达95%；建成北疆医学中心和健康城市，市民主要健康指标达到或接近发达国家和地区水平；新建改建各类保障性住房1万套，居住条件居全国前列；养老服务设施覆盖全部社区，人均期望寿命达到81岁；城镇登记失业率控制在4%以内；民生保障水平居全国前列。

——安全基础更加牢固。安全生产事故总量进一步下降，事故死亡率、受伤人数、经济损失总体呈下降趋势，食品药品安全监管形成严密高效、社会共治的治理体系，全社会安全生产的基础进一步夯实；新建居民小区各族市民嵌入式居住率、民汉家庭结对率100%；零发案社区实现全覆盖；公众安全感指数达到90%以上，蝉联“全国最安全城市”称号。

围绕上述目标，将在统筹推进各项工作的基础上，着力抓好四个方面的工作：

（一）全力构建多元外向的现代产业体系。

以六大基地建设为支撑，打造丝绸之路经济带核心区的石油中心。全力支持油田企业加大勘探攻关力度，落实规模优质储量，确保2021年油气当量1450万吨，实现辖区原油年产量1000万吨。积极支持社会资本参与油气上游开发，引导油气资源加工企业在高新区聚集，实现国内外两个渠道引入原料资源。推动新疆油田公司和独山子石化公司完成属地注册与合资合作，加快推进独山子石化公司炼油轻质化及乙烯优化调整项目。启动克拉玛依石化公司超稠油加工技术改造项目，建成高品质润滑油和高等级道路沥青两个百万吨基地。到2021年，力争全市炼化能力达2000万吨。加快推进科学城建设，建设中亚地区最具影响力的石油科技研发中心。充分发挥新疆石油工程技术服务企业联合会作用，支持地方综合油气服务集团“走出去”，推动油田服务向外辐射。发挥亚欧博览会和石油装备展平台作用，加快建设面向中亚的机械制造和装备技术服务中心，打造全覆盖的石油设备制造产业链，建成机械装备制造出口产业集聚区。积极参与国家油气管道建设，打造国家能源大通道上重要的油气储备基地。支持中国石油大学（北京）克拉玛依校区重点实施应用型本科、专业研究生培养，扩大留学生培养，引领带动克拉玛依职业技术学院、克拉玛依技师培训学院，打造应用型区域石油石化人才培养基地。

以三大新兴产业发展为依托，打造丝绸之路经济带核心区的信息中心、区域能源金融平台和区域旅游集散地。建设国家级云计算产业园，积极争取将克拉玛依“丝绸之路经济带信息中心”建设纳入国家战略布局，进一步扩大园区数据中心规模和能力，争取国家部委、援疆省市数据灾备中心等项目落地。大力推动影视渲染、跨境电子商务、多语种信息资源加工处理等产业发展，打造面向丝绸之路经济带的数据存储、数据处理和应用服务基地。支持昆仑银行、中石油专属财产保险公司、金龙国民村镇银行等地方法人金融机构加快发展，积极促进国有商业银行、保险、证券等金融机构加大产品开发力度，发展股权投资基金、保理、租赁等新兴金融业态，更好地助力实体经济发展。支持新疆中亚商品交易中心拓展线上业务，努力建成国家综合性石油交易中心。到2021年，新增5家企业在A股市场上市，1家企业在海外上市，力争25家企业在全国股转系统挂牌。加快魔鬼城5A级景区创建、西戈壁地质公园、世界石油博览园等重点旅游项目建设，重点布局克拉玛依城市休闲旅游核心区、独山子天山自然与人文旅游区、乌尔禾自然观光与摄影书画旅游区、白碱滩石油文化旅游区、农业开发区田园生态观光旅游区建设，到2021年，旅游人数突破680万人次。信息、金融和旅游业年均增长分别达30%、20%、18%以上。

以创新为驱动，打造驱动产业发展的创新服务体系。加快科学城、大学城、高新园区、云计算产业园“两城两园”建设，用好与大连化物所共同搭建的“一基地两中心”科技创新平台，形成石油石化产业、信息产业、金融产业的标志性科技成果和行业核心知识产权。积极将高新区创建为国家高新区和全国产城融合示范区，以超常力度和举措集聚创新创业所需的各类要素资源。落实科技成果转化等创新激励政策，探索央企科技成果向地方企业有偿转让的市场机制。支持国有企业、科研院所管理人员和科技骨干人才离岗创业、兼职取酬、创办领办企业。重点引进一批高层次人才和技术领军人才，培养一批产业发展急需的技能型、实用型人才。

（二）努力建设开放文明的高品质城市。

紧紧围绕打造高品质城市的目标，通过打造一流的教育、一流的医疗、优美的环境和现代化的管理，让克拉玛依的“软硬件”水平再上新台阶。深化教育领域综合改革，重点实施未来教育家培养工程和卓越教师培养计划，建设一流的教育人才队伍，拓展和提升中外合作办学项目，加速教育现代化进程，逐步建成中亚地区一流的教育和具有国际竞争力的人才培养高地。推动北疆医学中心和健康城市建设，市民主要健康指标达到或接近发达国家（地区）水平。积极推进生态文明，抓好城市周边荒漠生态恢复、古海湿地公园、金龙湖生态景观等项目建设，全市绿地面积达到12万亩，环境空气质量优良率达91%以上。倡导低碳生产、生活方式和绿色消费理念，落实节能减排措施，推进资源节约型社会建设。进一步完善城市功能，推进综合医院、中亚国际学校、城市综合管廊等一批基础设施建设工程，新建、改建各类保障性住房1万套。打造四位一体的区域综合交通运输体系，推动克拉玛依国际机场、乌鲁木齐—克拉玛依城际高铁、克拉玛依—阿拉山口和吉木乃口岸高等级公路建设，争取新开通3—5条直飞内地一线城市的航线，开通1条国际航线并实现商检、边检和海关落户。实现铁路客运量200万人次、航空客运量100万人次。深入推进城市管理执法体制改革，全面建成权责明晰、协调联动、多元共治的城市管理“大联动”机制，实现城市管理精细化、规范化、专业化。弘扬多元融合的克拉玛依文化，传承“克拉玛依精神”，蝉联“全国文明城市”称号。

（三）着力打造和谐稳定的最安全城市。

（四）全面提升政府治理体系和治理能力现代化水平。

加快建设法治政府、务实政府、服务政府、廉洁政府，增强政府的执行力和公信力，努力为各族群众提供优质高效的服务。深入贯彻依法治国基本方略，将政府工作全面纳入法治化轨道，用法治思维和法治方式履职尽责。以全面深化改革为契机，加大行政管理体制改革力度，加快推进职能转变和简政放权，提升政府服务管理水平。完善监管体制，创新监管方式，提高监管效能，充分释放市场和各类主体发展活力，促进经济社会和谐发展。

2017年的重点工作建议

主要发展目标：

地区生产总值（GDP）增长5%；扣除中央石油石化企业贡献份额，地方生产总值增长10%；第三产业增长10%；全社会固定资产投资增长50%；一般公共预算收入保持正增长；社会消费品零售总额增长10%；居民消费价格指数涨幅控制在3.5%以内；城镇登记失业率控制在3%以内；城镇居民人均可支配收入与自治区保持同步增长；努力完成节能减排降碳任务。

根据发展目标，突出抓好以下工作：

（一）以创新举措力推传统产业改造升级。

油气生产和炼油化工方面，支持油气生产企业突出技术和管理创新，深化提质增效，用创新促进油田降本增效。推动新疆油田公司、春风油田、富城能源等多元油气开发主体，加大辖区勘探开发力度，扩大生产规模，力争全年实现原油产量1215万吨，天然气产量29.3亿立方米。力争年内完成新疆油田公司属地注册与合资合作改革。以新疆推进能源综合改革示范区为契机，支持社会资本参与油气上游开发，加快企业整合，增强核心竞争力。

积极争取中石油集团公司加快推进独山子石化公司炼油轻质化及乙烯优化调整项目，启动克拉玛依石化公司超稠油加工技术改造项目，形成新的经济增量。两大石化公司争取原油加工量1300万吨。力争今年完成独山子石化公司属地注册与合资合作改革。鼓励驻市央企给予地方企业更多支持，解决地方炼化企业原料供应和原料品质问题，争取天利实业芳烃综合利用项目落地，最大限度地延伸炼化下游产业链。全力推进高新区创建国家级高新区工作。

技术服务和机械制造方面，加快推进科学城建设，聚集科研院所和人才，打造城市创新发展的重要平台。发挥新疆石油工程技术服务资质认定中心作用，为地方企业走出去提供资质服务。加快城市物流保税区建设，支持跨境电商业务加速发展，吸引周边地区更多货物在锦泰国际物流园集聚和通关。

围绕中国制造2025，拓展炼化装备制造、钻采装备制造、地面装备集成、移动式作业设备等产业，形成分工协作的石油装备产业链。加快机械产品质量检测认证中心和石油机械装备技术研究中心组建步伐。积极支持与三一集团务实合作发展，推进智能研发、智能制造，提升装备制造业整体水平。办好第四届中国（克拉玛依）国际石油天然气及石化技术装备展览会。

油气储备和工程教育方面，积极争取中央石油企业新的石油战略储备和石化成品油商业储备项目落户，提升储备能力，服务国家能源安全战略。

完善中国石油大学（北京）克拉玛依校区基础设施建设和办学条件建设，与校区共建金融学院，为地方经济发展提供人才支撑。推动工程教育基地各类资源实现共享共赢，探索“政企学研”合作模式，引入更多创新元素，与地方企业合作实现产业化，助力城市创新发展。进一步扩大克拉玛依职业技术学院、技师培训学院招生规模，提升教育教学水平。以面向国内外市场开展石油石化专业技术、多语种培训和其他专业人才培训为主，尽快扩大培训规模和能力。

（二）以开放理念力促新兴产业加快发展。

信息产业方面，依托新疆“天山云”和北疆西部区域融合发展平台，加快创建国家大数据综合试验区，开展云计算产业园软件园二期建设工作，力推中国电信（新疆）数据中心、中国移动（新疆）数据中心（二期）建设，争取更多第三方数据中心和国家部委、援疆省市备份业务落户我市，形成数据中心的规模优势。推动克拉玛依到国内主要城市的链路建设，提高链路质量，降低链路价格，进一步拓展云服务输出能力。加快推进“一带一路”影视动漫渲染基地等项目实施，打造新疆特色数字文化产业链，汇聚更多应用企业落户园区。支持油城数据公司发展，支持政务云、医疗服务、食品溯源等智慧成果产品向周边地区全面推广，形成可推广的信息系列化产品，提高信息产业效益。信息产业增加值力争增长30%以上。

金融产业方面，支持天利高新新业务发展和中石油金融板块落户克拉玛依。支持昆仑银行设立喀什分行、中石油专属财险公司设立新加坡分公司。推动科力新技术、丝路金音等企业在主板上市，新增1—2家企业在全国股转系统挂牌。支持中亚商品交易中心扩大线上销售产品品种和规模，打造新疆油气交易中心。探索金融创新支持实体经济发展的政策措施，积极引进银行、证券、保险、融资租赁、股权投资等各类金融主体，不断丰富金融业态，增大金融体量，扩大有效供给，提升金融服务能力。金融产业增加值力争增长20%以上。

旅游产业方面，推进首批国家级旅游业改革创新先行区工作，激发旅游市场活力。整合乌尔禾旅游资源，重点推进魔鬼城5A级景区创建和 “中国摄影书画小镇”建设，打造文化旅游影视基地。加快田园生态旅游区建设，拓展农业体验、采摘、休闲、科普、养生等服务功能，推动农业开发区特色生态旅游发展。大力发展自驾游、户外运动产业，利用全国性赛事资源，延伸挖掘汽车后产业项目，不断丰富旅游新业态。旅游业收入力争增长25%以上。

（三）以包容精神增强城市发展活力。

发展壮大地方企业。围绕供给侧结构性改革，以提高质量和效益为中心，加快推进地方国有企业改革，培育具有较强竞争实力和外向发展能力的龙头企业，努力构建央企和地方国企紧密关联、相互嵌入发展的新格局。理顺供水、供电、供暖、供气企业运营管理机制，完善城市功能。强化领导干部联点帮扶企业工作机制。大力推动和支持民营经济多元创新发展，提升企业核心竞争力，加快培育行业领军企业。完善“政银企”合作平台，发挥“助保贷”和“科易贷”资金池的作用，引导融资担保机构支持民营企业和小微企业的转型发展，切实解决企业融资难题。

强化招商引资工作。在现有政策的基础上，研究制定有吸引力的招商引资优惠政策，通过创造良好的政策环境，吸引更多企业到克拉玛依投资兴业。发挥商会协会作用以商招商，紧抓国家和自治区鼓励产业发展政策，重点关注化工新材料、碳纤维材料、信息互联网、智能制造、节能环保等科技前沿技术领域，提升招商引资质量，促进产业向价值链高端延伸。全年力争实现招商引资到位资金131亿元，增长54%。

推进现代农业发展。立足打造田园生态农业目标，抓好有机瓜菜种植基地建设，增加绿色优质农产品有效供给。支持乌尔禾镇、小拐乡农业健康发展，加大农业开发区产业加工区招商力度，创新发展“四地五师”农产品加工园，推动区域融合及产业合作，实现向生态化、现代化、创新化方向发展。

发挥科技创新引领作用。着力打造高新区创新引擎，以与大连化物所共建“一基地两中心”、与三一集团共建克拉玛依石油工艺与智能装备研究院为平台，以中国石油大学（北京）克拉玛依校区为依托，加快创新基地建设步伐，不断聚集创新要素和资源，催生发展新动能，构建起以企业为主体的“产学研用”协同创新体系，让创新成为推动工作、促进发展的第一动力。全力支持中国石油大学（北京）建设油气资源与探测、重质油两个国家实验室克拉玛依分室，全力支持企业共建院士工作站，进一步提升科技创新水平和对经济增长的贡献度。加快制定科技成果转化等创新激励政策，探索央企科技成果向地方企业有偿转让的市场机制，鼓励科技人才以多种方式支持地方企业科研工作。利用援疆省市科技创新资源及人才资源，搭建人才引进与培养平台。

深化对外交流合作。强化与丝绸之路经济带和中亚经济走廊沿线更多重要节点城市建立友好关系，广泛开展交流合作。组织我市企业参加境外的石油天然气展会，带动更多地方企业“走出去”拓展外部市场。举办好丝绸之路经济带新疆克拉玛依论坛、丝绸之路科学节、旅游商品博览会、北疆西部区域融合发展研讨会等一系列展会活动，力争办出品牌，为我市的开放发展搭建交流合作平台。

（四）以人民为中心打造高品质城市。

增强城市规划引领作用。优化城市空间布局和形态，打造生产空间集约高效、生活空间宜居适度、生态空间天蓝水秀的良好环境。总结经验、梳理项目，启动中国人居环境范例奖申报。加快推进高新区、云计算产业园区总体规划修编，构建有效支撑可持续发展的保障体系。强化地方性建筑材料、绿化植物的研究应用，高水平设计城市，塑造现代城市特色。优化商业布局模式，培育发展特色商业街区，完善社区商业便利消费体系规划和建设。

加快重点项目建设。推进工程教育基地、厚博学院、综合医院等重点项目建设，扎实做好智慧城市、地下综合管廊建设前期工作，统筹“四供”移交业务与城市功能的衔接，不断完善供电供水、排水防涝、垃圾污水收集处理等市政基础设施。按照国家产业政策和投资方向，全力争取将克拉玛依机场改扩建、乌鲁木齐—克拉玛依城际高铁、克拉玛依—阿拉山口公路、风城水库—三平水库输水管道等重大项目列入国家、自治区重点建设计划，力争获得更多政策和资金支持。积极引入社会力量参与基础设施建设。

切实做好生态环境建设。落实“大绿化”工程建设任务，重点完成金龙湖、独山子城市公园等26项绿化任务，城市建成区全部实现绿化。加强城市外围生态环境建设，编制和完善戈壁生态建设规划，将中心城区西北部区域优先进行封育，逐步将其调整为林地并将重点区域划为自然保护区，加强监管和保护。强化节能减排降碳目标责任落实，积极参与新疆碳排放交易市场建设。在做好自治区第一批低碳城市、低碳社区试点工作的基础上，争取将我市申报为国家级低碳城市试点。持续开展“蓝天工程”建设，发挥好奎—独—乌区域大气污染联防联控机制，力争全市空气质量优良率达到91%以上。高度重视水资源集约节约利用，吸引社会资本参与古海湿地公园、艾力克湖等生态型水源管理，实现旅游开发与水源保护协调推进，实现水资源效益的最大化。

持续推进城市精细化管理。以智慧城市建设为依托，以诚信体系建设为抓手，强化城市建设管理重心下移，做实城市管理大联动机制，加强城市管理的协调、监督和考核，不断提高精细化管理水平。健全违法建设治理机制，强化拆违主体责任，全面加强源头防范，完成存量违建处置，坚决杜绝新增违建，切实巩固城市管理顽症整治成效。

力促服务业发展。积极支持和引导生活性服务业发展，落实好扩大内需、拉动消费政策，推动供需结构有效匹配，满足市民多层次消费需求。着力培育健康养老、家政服务、教育培训、文化体育等新的消费热点。提升国际家居建材城、国际汽车城、西域玉都等园区的专业品质，努力化解商业房产库存，推动建立“线上服务＋线下体验”、“网络订购＋实体配送”等多业态互补的运营模式，延长产业链，提高专业化市场的聚集辐射能力。

（五）以为民情怀落实惠民举措。

坚持就业惠民。着力拓宽就业渠道，积极搭建就业创业平台，强化企业作为就业主阵地的积极作用，鼓励引导高校毕业生、就业困难群众等多渠道就业创业，提高全社会就业能力和就业质量，通过就业吸引创业，通过创业培训带动就业。全年发布就业岗位5000个以上，实现就业再就业3000人，新增创业人数500人，“零就业家庭”动态为零。引进和培养一批高层次人才、技术领军人才和创新型人才，助力产业和经济社会发展，以产业发展促进就业增长。

坚持教育惠民。切实提升教育内涵和价值导向，深化教育“管办评”改革，加速推进现代学校制度建设，深层次学习国内外先进教育理念、办学经验，推进教育国际化、现代化进程。加强教师队伍建设，全面实施全市学前三年免费教育，扎实推进“统一编班”工作。

坚持医疗惠民。全面推进公立医院改革，鼓励支持社会办医，落实公立医院管理自主权。强化重大传染病和慢病防控措施，提升公共卫生服务水平和突发公共卫生事件应急处置能力，增强大健康观念。巩固完善基层卫生运行机制，健全家庭医生签约服务机制。全面落实两孩政策，有效控制政策外违法生育现象。积极对接医疗人才“组团式”援疆，以厚博学院和综合医院为载体，构建“医教研”协同型卫生服务体系，不断提高健康服务水平。

坚持社保惠民。推进全民参保计划，推动社会保障由制度全覆盖向法人全覆盖转变。落实全民健康体检计划，建立居民意外伤害保险制度，持续推进异地就医结算，扩大结算范围，进一步完善多层次的社会保障体系。做好社保基金结构调整优化，评估并调整应对经济下行和支持实体经济发展降费的政策措施。加快推进养老服务业发展，建立健全三位一体的养老服务体系，推进医养结合。不断完善社会救助体系，建立居民家庭经济状况核对机制，逐步实现救助精准化。

坚持安居惠民。将保障房规划建设与城市长远发展相结合，新建公租房201套，完成棚户区改造442套，确保新就业职工和外来务工人员住有所居。稳妥推进第七师奎东农场、第八师136团棚户区项目改造工作。调整住房保障和供应结构，积极探索限价商品房建设。发挥物业管理服务四级监督管理网络作用，为市民提供更加优质的物业管理和服务。

坚持暖心惠民。扎实做好对口支援喀什“一市两县”的协作扶贫，确保完成阶段性扶贫攻坚任务。继续优化调整公交路线，重点解决城南新区居民乘车难问题。积极推动智慧城市惠民工程，研发便民服务平台，促进信息消费产业发展，为市民提供优质服务。

坚持文化惠民。以国家公共文化服务体系示范区建设为抓手，打造区域性公共文化数字综合服务平台，拓展市图书馆、文化馆等场馆体验功能，做好群众喜闻乐见的文化活动，抓好城市生日庆典、新春音乐会、诗书会、水节、摄影书画等特色活动，进一步丰富内涵，把文化活动办出特色，持续提升城市文化内涵和市民文化素养，让文化滋养克拉玛依人的精神世界。

坚持安全惠民。突出民生导向，在重点行业、重点领域持续深入开展反垄断与反不正当竞争执法、“红盾护农”、网络市场监管等专项行动，严厉打击不正当竞争、侵权假冒等违法行为。强化餐饮食品全过程监管，不断提升食品药品检验检测能力，确保全年无重大食品药品安全事故。严格落实安全生产“四严”要求，加快推进风险分级管控和隐患排查治理双重预防性机制建设，强化重大隐患治理和安全专项整治，坚决遏制重特大安全生产事故发生，确保全市安全生产形势持续稳定。

（六）以责任担当全力维护社会稳定。

牢牢把握维护社会稳定的主动权；牢牢占领意识形态领域的主阵地；筑牢长治久安的群众基础；筑牢社会治理创新的基层基础。

（七）以简政放权强化政府自身建设。

严格依法行政，建设法治政府。完善依法行政制度体系，为改革决策提供保障。进一步完善科学民主依法决策机制，健全行政执法体制，强化政府法律顾问、公职律师体系，规范执法程序，严格按照法定程序和权限管理经济社会事务。自觉接受人大法律监督和政协民主监督，认真听取民主党派、工商联及各人民团体意见。深入推进政务公开，广泛接受社会公众和新闻舆论监督。强化政府诚信体系建设，不断提高政府公信力。

切实转变作风，建设务实政府。巩固和扩大群众路线教育实践活动、“三严三实”专题教育、“两学一做”学习教育成果，加强政风行风建设，认真解决乱作为、不作为、慢作为等问题，坚决纠正部门和行业不正之风，努力树立勤政高效、清正廉洁的政府形象。

持续简政放权，建设高效政府。按照“一级政府、一级财力、一级事权”的原则，进一步推进简政放权，重点扩大区级政府在投资建设、城市管理、行政执法、市场监管等领域的事权。深化机构改革，组建城市管理委员会、住房和建设管理局，探索推进高新区与金龙镇、克拉玛依区与农业综合开发区管理体制改革。建立健全行为规范、运转协调、公正透明、廉洁高效的行政管理体制，严格落实工作责任制，强化督办考核，探索建立正向激励机制，激发公务人员工作的积极性、主动性，保持高效率、快节奏的工作状态，确保各项目标任务落到实处。

推进反腐倡廉，建设廉洁政府。严格落实中央八项规定和党风廉政建设责任制、党员廉洁自律准则、领导干部廉洁从政准则等党纪党规。坚持对腐败“零容忍”，严肃财经纪律，牢固树立过“苦日子”的思想，进一步降低行政成本，规范招投标、土地征收出让、公共资源和国有资产转让等行为，打造廉洁政府。

各位代表，重任在肩，使命光荣，实干才能实现梦想。让我们紧密团结在以习近平同志为核心的党中央周围，在自治区党委和市委的坚强领导下，进一步解放思想、敢于担当，为推进世界石油城建设，实现克拉玛依社会稳定和长治久安而努力奋斗！
